# Synthetic scaffolds help airway cells reach maturity

**DOI:** 10.7554/eLife.21786

**Published:** 2016-11-01

**Authors:** Marko Z Nikolić, Emma L Rawlins

**Affiliations:** Wellcome Trust/CRUK Gurdon Institute, University of Cambridge, Cambridge, United Kingdommn340@cam.ac.uk; Wellcome Trust/CRUK Gurdon Institute, University of Cambridge, Cambridge, United Kingdome.rawlins@gurdon.cam.ac.uk

**Keywords:** organoids, lung airway, human pluripotent stem cells, transplantation, scaffolds, Mouse

## Abstract

Transplanting bioengineered human lung organoids into mice could lead to a humanized model for pre-clinical studies of lung disease.

**Related research article** Dye BR, Dedhia PH, Miller AJ, Nagy MS, White ES, Shea LD, Spence JR. 2016. A bioengineered niche promotes in vivo engraftment and maturation of pluripotent stem cell derived human lung organoids. *eLife* **5**:e19732. doi: 10.7554/eLife.19732

The human airways bring air into and out of the lungs, and the epithelium that lines these airways uses a combination of mucus and hair-like structures called cilia to clear inhaled particles and microorganisms from the lungs. For decades cell biologists have been able to make mature airway epithelia *in vitro* by taking airway cells from patients and cadavers and growing them in culture. These epithelia have been used for a range of applications, from fundamental cell biology and cancer genetics to clinical diagnosis and pharmacological testing (discussed in Fulcher et al., 2005). In recent years researchers have been trying to use human pluripotent stem cells – cells that can differentiate to become any of the types of cells that are found in the body – to make mature airway epithelia. However, if such epithelia are available from real people, potentially with a complete medical history, why is there any interest in using pluripotent stem cells to make them?

The answer is two-fold. First, the adult epithelia grown in culture suffer from various limitations: they are highly variable, they contain only a limited range of cell types, and it is not possible to introduce targeted genetic mutations in them, although numerous labs are working to solve these issues (Mou et al., 2016; Butler et al., 2016). Second, the use of pluripotent stem cells to make mature airway epithelia has the attraction of allowing researchers to study the progressive development of a disease over time. In addition, the use of pluripotent stem cell lines will make it possible to introduce a specific genetic alteration and study its effect by comparing these cell lines to otherwise genetically identical control cells. Reproducible growth of epithelia with these features has the potential to improve disease modeling, to facilitate drug discovery across a range of lung diseases, and to provide a limitless supply of cells for toxicology testing, or even for regenerative medicine. However, while there have been numerous reports of methods for producing mature airway epithelia from pluripotent stem cells, the cells in these epithelia have tended to resemble embryonic and neonatal cells rather than mature adult cells.

The airways and lungs are derived from a region in the developing embryo called the anterior foregut endoderm. A typical method for making mature epithelia from stem cells involves starting with pluripotent stem cells and coaxing them to become endoderm cells, then anterior foregut endoderm cells, then lung progenitor cells (which can become any type of lung cell) and, finally, various types of airway epithelial cells. One recent innovation involved the growth of three-dimensional structures called human lung organoids by a group at the University of Michigan (Dye et al., 2015) and another group at Kyoto Univerisity and Osaka University (Konishu et al., 2016). These organoids contained many of the cell types that are found in mature airway epithelia but, notably, they did not contain mature goblet cells (the cells that produce mucus) or a number of other secretory cells (Figure 1A). Now, building on their previous work, Jason Spence of the University of Michigan and co-workers – including Briana Dye as first author – report that these shortcomings can be overcome by using a combination of synthetic "scaffolds" and *in vivo* growth in mice (Dye et al., 2016).

**Figure 1.**
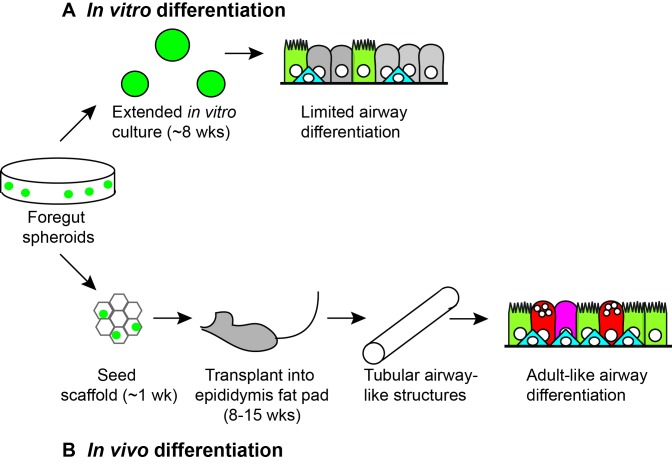
*In vitro* and *in vivo* differentiation of lung organoids. Human pluripotent stem cells (not shown) can be differentiated to become three-dimensional bunches of cells that are fated to anterior foregut endoderm cells. When Dye et al. plated such foregut spheroids (green circles) into a substrate (Matrigel) and cultured them *in vitro* for approximately 8 weeks (A), they observed levels of differentiation similar to embryonic human lungs, including ciliated cells (light green), basal cells (blue) and rare secretory cells (not shown). However, when Dye et al. seeded foregut spheroids onto synthetic scaffolds and cultured them for approximately 1 week *in vitro*, and then transplanted them into the epididymal fat pad of immunocompromised mice for 8–15 weeks (B), they observed greater levels of differentiation, including ciliated cells (light green), basal cells (blue), goblet cells (red) and secretory cells (magenta) lining the surfaces of tubular airway-like structures. The organoids also appeared to be connected to the vasculature of the mice.

It has become a standard technique to implant immature organoids (or cells derived from pluripotent stem cells) into well-vascularized sites in mice whose immune system has been compromised. The growth of the cells in this *in vivo* environment, typically in the kidney capsule or in fatty tissue adjacent to the stomach or epididymis, helps them to mature properly, presumably due to the physiological levels of nutrients, oxygen and circulating factors. Dye et al. started by taking human lung organoids that had been grown in a basement membrane substrate (Matrigel) and implanting them directly into the mice. Although human epithelia were recovered from these initial transplants, they rarely contained lung cells.

In an attempt to solve this problem Dye et al. decided to first culture the organoids on synthetic scaffolds made of a polymer called Poly(lactide-co-glycolide), which is used in medical devices and for culturing pancreatic cells (Yap et al., 2013). Given the size of the scaffolds, the only suitable transplantation site was the fat pad adjacent to the epididymis (which is near the testicles in male mice). When the scaffolds were retrieved from the mice, typically following 8–15 weeks of growth *in vivo*, the organoids had frequently grown into tubes that resembled the adult airway and, moreover, they contained a wider range of mature cell types than had been produced before (Figure 1B). Importantly, this latter finding was validated by detailed comparison to human fetal and adult airways.

The *in vivo* system developed by Dye, Spence and co-workers appears to be reproducible across multiple pluripotent cell lines and could be used to develop improved models of adult lung diseases and for pre-clinical drug testing. And if we can learn more about the mechanisms that underlie the maturation process, it might be possible to produce mature epithelia *in vitro* for high-throughput (drug discovery-type) experiments. It will also be important to assess whether mature alveoli (the lung cells that are involved in the exchange of oxygen and carbon dioxide) can be generated from pluripotent stem cells through similar *in vivo* transplantation approaches. Further study is also needed to assess how different types of scaffolds affect the maturation of cells *in vivo*.
